# The preventative effects of *Lactococcus Lactis* metabolites against LPS-induced sepsis

**DOI:** 10.3389/fmicb.2024.1404652

**Published:** 2024-07-17

**Authors:** Yue Fu, Song Zhang, Qiulin Yue, Zaiyong An, Minghan Zhao, Chen Zhao, Xin Sun, Kunlun Li, Baojun Li, Lin Zhao, Le Su

**Affiliations:** ^1^State Key Laboratory of Biobased Material and Green Papermaking, School of Bioengineering, Qilu University of Technology, Shandong Academy of Sciences, Jinan, China; ^2^Shandong Baoyuan Biotechnology Co., Ltd., Jinan, China; ^3^Shandong Chenzhang Biotechnology Co., Ltd., Jinan, China; ^4^Shengsheng Xiangrong Biotechnology (Shandong) Co., Ltd., Jinan, China

**Keywords:** sepsis, *L. lactis*, gut microbiota, preventive effects, chip analysis

## Abstract

**Introduction:**

Sepsis is a syndrome of organ dysfunction caused by a dysregulated host response to infection and septic shock. Currently, antibiotic therapy is the standard treatment for sepsis, but it can lead to drug resistance. The disturbance of the gut microbiota which is affected by sepsis could lead to the development of organ failure. It is reported that probiotics could shape the gut microbiota, potentially controlling a variety of intestinal diseases and promoting whole-body health.

**Methods:**

In this study, we evaluated the preventive effects of intra- and extracellular products of probiotics on sepsis. The extracellular products of *Lactococcus lactis* (*L. lactis*) were identified through the *in vivo* cell experiments. The preventive effect and mechanism of *L. lactis* extracellular products on mouse sepsis were further explored through HE staining, mouse survival rate measurement, chip analysis, etc.

**Results:**

*L. lactis* extracellular products increase cell survival and significantly reduce inflammatory factors secreted in a cellular sepsis model. In *in vivo* experiments in mice, our samples attenuated sepsis-induced pulmonary edema and inflammatory infiltrates in the lungs of mice, and reduced mortality and inflammatory factor levels within the serum of mice. Finally, the mechanism of sepsis prevention by lactic acid bacteria is suggested. Extracellular products of *L. lactis* could effectively prevent sepsis episodes.

**Discussion:**

In animal experiments, we reported that extracellular products of *L. lactis* can effectively prevent sepsis, and preliminarily discussed the pathological mechanism, which provides more ideas for the prevention of sepsis. In the future, probiotics may be considered a new way to prevent sepsis.

## 1 Introduction

Sepsis is a serious, potentially fatal, organic dysfunction caused by a dysregulated host response to infection and septic shock in a subset of patients in which underlying circulatory and cellular/metabolic abnormalities are sufficiently profound to substantially increase mortality (Jiang L. et al., 2022). Extrapolating from the study data, there may be 5.3 million deaths from the 31.5 million cases of sepsis worldwide (Markwart et al., 2020). In a recent study performed in Brazil, one-third of intensive care beds were occupied by septic patients, with a mortality rate of 55.7% (Kahveci et al., 2021). Early identification of patients with sepsis, early intravenous fluid resuscitation, and early intravenous antibiotic administration are the mainstay of sepsis management (Aljerian et al., 2019). Despite advances in clinical management, including the findings of new biomarkers with diagnostic and prognostic value, sepsis and septic shock still remain a leading cause of admission to intensive care unit worldwide (Colomina-Climent et al., 2016). The appropriate and timely administration of antibiotic therapy play an essential role in the achievement of a favorable outcome for patients with sepsis and septic shock, but the alarming and rising phenomenon of antibiotic resistance among the causative pathogens may represent the most serious challenge for future years if new drugs will not be found (Bogner et al., 2013; Messina et al., 2015).

More than 95% of sepsis is caused by bacterial infection, and the release of endotoxin lipopolysaccharides (LPS) caused by bacterial infection is the main factor leading to sepsis (Sun et al., 2021). When the body is invaded by LPS, Toll-like receptor 4 on the surface of macrophages recognizes LPS and activates intracellular signal transduction of macrophages to produce a large number of pro-inflammatory cytokines, such as TNF-a, IL-1β, IL-6, IL-12, etc. Cytokines reach distal target organs or target cells through blood circulation or paracrine and exert biological effects, causing systemic inflammatory reactions and leading to sepsis. The endothelium is an active contributor to sepsis and as such represents a major target for therapy. During sepsis, endothelial cells amplify the immune response and activate the coagulation system (Yang et al., 2011). They are both the target and source of inflammation and serve as a link between local and systemic immune responses (Shin et al., 2013). By reversing and maintaining the function and status of endothelial cells, it will play a positive role in the protection of the pathogenesis center of sepsis, liver, spleen, lung, kidney and other important organs (Liu et al., 2019; Fu et al., 2023).

Probiotics, which include bacteria and yeast, are living microorganisms that have been shown to be beneficial to human health. Clinical trials and *in vivo* experiments have expanded our current understanding of the important role that probiotics play in human gut microbiome related diseases. Many clinical trials have demonstrated that probiotics could shape the gut microbiome, potentially controlling a variety of gut diseases and promoting overall health (Ahn et al., 2020). For decades, the gut was thought to play an important role in sepsis pathogenesis. The composition of the intestinal microbiome, is affected by sepsis, and might contribute to the development of organ failure (Hu et al., 2019). A well-balanced microbiota is essential to maintain enteric and systemic immune homoeostasis. Disruption of the integrity of the intestinal microbiota potentially increases susceptibility to sepsis (Deng et al., 2023). Therefore, probiotics can reduce sepsis symptoms by reducing intestinal inflammation and repairing intestinal function in patients with sepsis. Every year, about 230 million surgical procedures are performed worldwide. Sepsis is one of the postoperative complications. Prophylactic antibiotic treatment is standard, but it is complicated by the rise of antibiotic resistance (Versporten et al., 2018). Studies have shown that the use of *L. plantarum*, *L. casei* and *B. breve* in combination with galactose oligosaccharide is also the best intervention for reducing sepsis, hospitalization and antibiotic use (van Ruler et al., 2011; Benjak Horvat et al., 2021). It is of great significance to prevent infection and improve sepsis through probiotics.

In this paper, LPS was applied to human vascular endothelial cells (HUVECs) coupling mice to induce sepsis. *Streptococcus lactis* (*S. lactis*), *Lactobacillus rhamnosus* (LGG), *Lactobacillus Paracasei* (*L. paracasei*), *Lactobacillus plantarum* (*L. plantarum*), Lactobacillus fermentium (L. fermentium) and *Lactococcus lactis* (*L. lactis*) were selected. The intracellular and extracellular products were extracted, respectively. Cell survival rate and cell inflammatory factors, mouse survival rate, mouse blood inflammatory factors, organ coefficient and microarray analysis were determined to analyze the effect of probiotics on sepsis.

## 2 Materials and methods

### 2.1 Cell culture

Human umbilical vein endothelial cells (HUVECs) were obtained as described (Michaux et al., 2006). Cells were cultured in M199 medium (Genview) supplemented with 20% fetal bovine serum (Applied Biosystems) in a humidified incubator at 37°C with 5% CO_2_. Cells were used in the 5th to 10th passages. For the HUVEC experiments, cells in logarithmic growth phase were seeded in well plate at a density of 1 × 10^5^ cells/ml. The cell morphology was observed by Nikon phase contrast microscope.

### 2.2 Culture of probiotics and preparation of extracellular and intracellular products

*Streptococcus lactis* (*S. lactis*), *Lactobacillus rhamnosus* (LGG), *Lactobacillus Paracasei* (*L. paracasei*), *Lactobacillus plantarum* (*L. plantarum*), Lactobacillus fermentium (L. fermentium), *Lactococcus lactis* (*L. lactis*) were inoculated on MRS solid medium (for the configuration of the medium, refer to Zhang et al., 2020) cultured at 37°C for 24 h, and then inoculated on their respective seed media, cultured at 37°C for 48 h, to obtain the seed liquid of the strain for use. The probiotic seed solution was inoculated in liquid medium for 24 h to obtain the bacterial solution, and the bacterial solution was precipitated after 3,000x g centrifugation for 15 min. After repeated rinsing of PBS for three times, the precipitation was collected by centrifugation and then decomposed by ultrasonic wave. Centrifuge 1,000x g for 10 min to collect the supernatant. After the bacteria were filtered by 0.45 μm filter membrane, the intracellular products of probiotics were obtained and stored in −80°C and liquid nitrogen, respectively. The probiotic seed solution was inoculated in liquid medium for 24 h to obtain the bacterial solution. The bacterial solution was centrifuged at 1000x g for 10 min to collect the supernatant. After the bacteria were filtered by 0.45 μm filter membrane, the probiotic extracellular products were obtained and stored in −80°C and liquid nitrogen, respectively.

### 2.3 Determination of cell viability

Cell viability was determined by MTT (3-[4,5-dimethylthiazol-2-yl]-2,5-diphenyltertrazolium bromide; M5655 Sigma-Aldrich) assay as previously described (Wang L. et al., 2020). The cells were seeded in 96-well plate. Cells were treated with 1, 2, 4 mg/mL intracellular or extracellular products of *Streptococcus lactis* (*S. lactis*), *Lactobacillus plantarum* (*L. plantarum*), *Lactobacillus fermentum* (*L. fermentium*) or *L. lactis* for 20 h or 44 h and then cells were incubated with 0.5% MTT for 4 h. After removing the medium, 100 μL dimethyl sulfoxide (DMSO) solution was added. The absorbance was measured at 570 nm using a SpectraMAX ABS microplate spectrophotometer (Molecular Devices). The cell viability was calculated by the ratio of OD.

Cell viability(%) = (sample group OD-blank group OD)/(control group OD-blank group OD) × 100%.

### 2.4 Enzyme-linked immunosorbent assays

The cells in logarithmic growth phase were seeded in 24-well plate. Firstly, HUVECs were pretreated with 2, 40, 800 μg/mL intracellular or extracellular products of *S. lactis, L. plantarum, L. fermentium, L. lactis* or MRS agar (MRS) medium for 12 h, 24 h or 48 h. Then, cells were cultured with 1 μg/mL LPS for 12 h. The cellular supernatant was obtained to determine the IL-6, IL-8 and TNF-α levels according to the manufacturers’ instructions (KE10007, KE10002 and KE10003, Proteintech Group, Inc.). Additionally, the serum of mice was collected to detect the IL-6 and TNF-α concentrations according to the instructions (Beijing dakowei Biotechnology Co., Ltd., Beijing, China).

### 2.5 Animal treatment

C57BL/6 mice aged 6 weeks (20 ± 2 g) were purchased from Beijing Vitonglihua Co., LTD. Mice were housed under standard conditions of humidity, room temperature and dark–light cycles. All animal experiments complied with the ARRIVE guidelines and were carried out in accordance with the U.K. Animals (Scientifc Procedures) Act, 1986 and associated guidelines, EU Directive 2010/63/EU for animal experiments and the National Institutes of Health guide for the care and use of Laboratory animals (NIH Publications No. 8023, revised 1978). The animal experimental protocol complied with the Animal Management Rules of the Chinese Ministry of Health (document no. 55, 2001) and was approved by the Animal Experiment Ethnics Committee of Qilu University of Technology. Mice acute sepsis model was established by intraperitoneal injection of LPS (20 mg/kg). The mice were divided into six groups: Mock group (NC), LPS group, LPS + MRS Group, low dose group (0.1 g/kg), medium dose group (0.2 g/kg) and high dose group (0.4 g/kg). At the beginning of the experiment, each animal in the mock and LPS groups was gavage 200 μL normal saline every day and each animal in the LPS + MRS group was gavage 200 μL MRS medium every day. Other animals were gavage 0.1 g/kg, 0.2 g/kg or 0.4 g/kg *L. lactis* extracellular products in the low-dose, medium-dose or high-dose groups every day. After 7 days treatment, except for the normal group, the mice in other 5 groups were intraperitoneal injected with LPS (20 mg/kg). The body weight of the mice was recorded every day and the physiological phenomena such as loss of appetite and blood in stool were observed.

### 2.6 Blood and tissue collection

The mice were euthanized by cervical dislocation. Serum was prepared by centrifugation at 3000 g for 20 min at 4°C and stored at −80°C for biochemical analysis. The organs of the mice were removed and the excess fat tissues at the edges of the organs were removed. After rinsing with normal saline at 4°C, the water was sucked up with filter paper and the organs were weighed. The lung was collected to obtain the wet weight, firstly. Then, the lung was dried at 80°C for 72 h to get the dry weight. Lung edema was assessed by calculating lung wet/dry (W/D) ratio.

### 2.7 Hematoxylin and eosin analyses

Lung tissues were fixed in an optimal cutting temperature compound (OCT) embedding medium (Tissue-Tek), frozen at −20°C for 12 h and then cut into 10 μm sections using a frozen sectioning machine (CM1950, Leica, Nussloch, Germany) and stained with hematoxylin and eosin (H&E) (Jiang K. et al., 2022). The sections were observed under an upright microscope (Eclipse E200, Nikon, Japan) and photographed using ImageView (Pooher, Shanghai, China).

### 2.8 Microarray analysis

Total RNA was extracted from cells treated with LPS alone for 24 h or cells treated the extracellular product of Lactococcus lactate and LPS for 24 h with TRIzol reagent (Invitrogen, United States). The samples were stored at −20°C, and microarray analysis was performed by CapitalBio Corp.^1^

### 2.9 Statistical analysis

All experiments were repeated at least 3 times independently. The normal distribution was firstly analyzed by SPSS v11.5 (SPSS Inc., Chicago, IL). All the data were expressed as mean ± SEM and analyzed with one-way ANOVA with use of SPSS v11.5 to compare the treatment means when data was normally distributed. The Tukey–Kramer multiple comparison procedure was used for Post-Hoc comparisons. *p* values <0.05 were considered statistically significant. Images were processed by the use of GraphpadPrism 5 (GraphPad Software, La Jolla, CA, United States) and Adobe Photoshop CS6 (Adobe, San Jose, United States).

## 3 Results

### 3.1 Effects of extracellular and intracellular products of probiotics on HUVEC morphology

**Figure 1:**
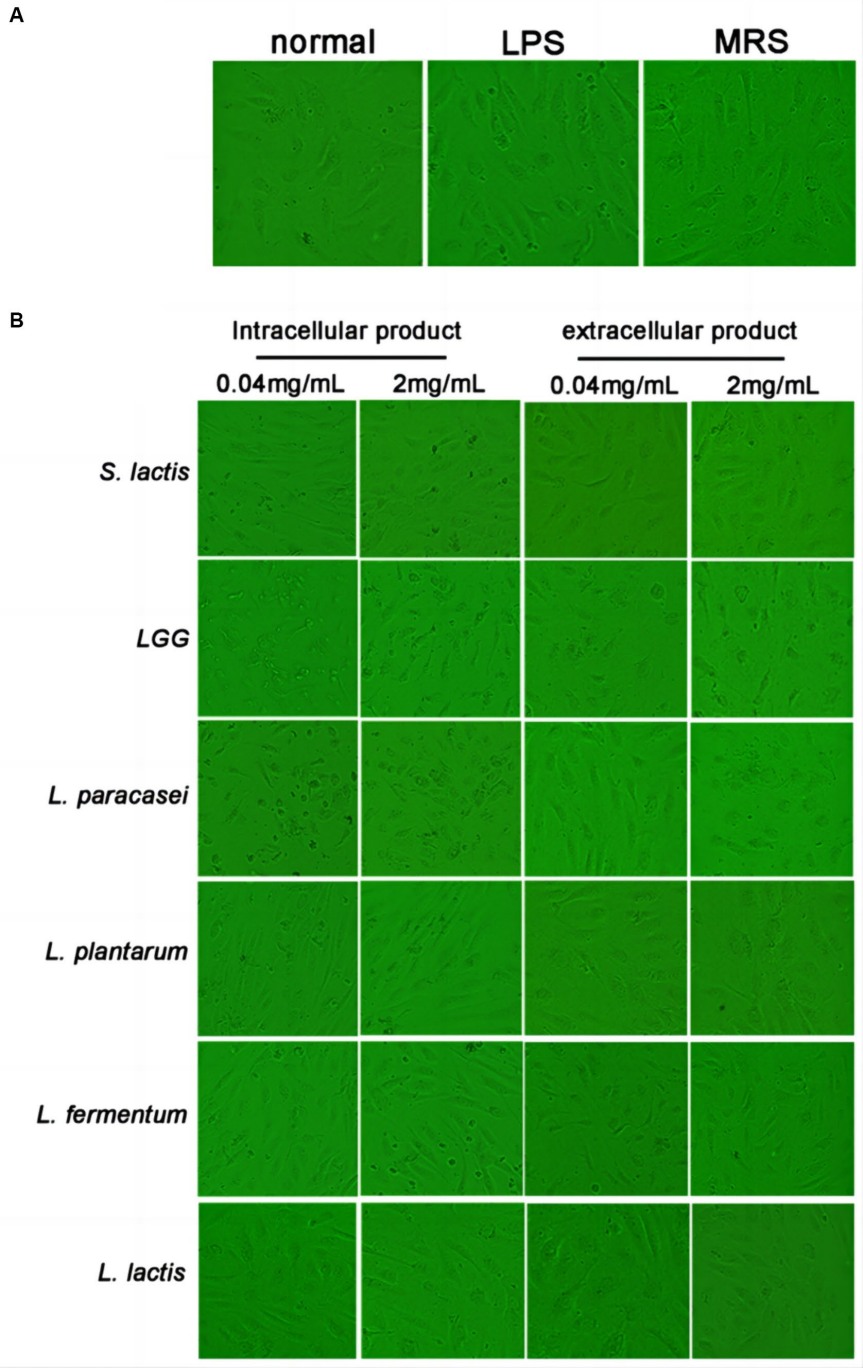
Effect of probiotic extracellular and intracellular products on the morphology of HUVEC. **(A)** The cell morphology was normal group, LPS group and MRS group. **(B)** Cell morphology of *S. lactis*, *LGG*, *L. paracasei*, *L. plantarum*, *L. fermentum* and *L. lactis* groups, respectively.

HUVECs were treated with 0.04 mg/mL or 2 mg/mL intracellular or extracellular products of *S. lactis*, *LGG, L. Paracasei, L. plantarum, L. fermentium or L. lactis*. The morphological changes of cells were observed after treated with 24 h. In the normal group, cells were full and most of them were attached to the dish with pebble-like morphology. The cells in LPS group were slightly rounded, the edges were not clear, and a small number of floating dead cells appeared. The cells in MRS group were in good condition, showing full adherence to the dish which were similar with cells in the normal group (Figure 1A). The number of cells treated with 40 μg/mL, 2 mg/mL, 4 mg/mL of LGG and L. paracase products decreased significantly. And most of the cells shrank and died (Figure 1B). Thus, at the same concentration, extracellular and intracellular products of *LGG and L. paracasei* showed more harmful to HUVECs. *S. lactis, L. plantarum, L. fermentum* and *L. lactis* were selected in the following experiments.

### 3.2 Cell viability assessment

MTT assay was used to investigate the cell viability. Cells were treated with the intracellular or extracellular products of *S. lactis*, *L. plantarum, L. fermentium* or *L. lactis* at 1 mg/mL, 2 mg/mL and 4 mg/mL for 24 h or 48 h. There were no differences between the intracellular products treated groups and normal group (Supplementary Figure S1). But after cells were treated with extracellular products of *S. lactis*, *L. plantarum, L. fermentium* or *L. lactis* for 48 h, cell viability was decreased (Supplementary Figure S2). Thus, the extracellular products greater than 4 mg/mL might be cytotoxic to HUVECs.

### 3.3 Effects of intracellular and extracellular products of probiotics on TNF-α, IL-6 and IL-8 levels in HUVECs

**Figure 2:**
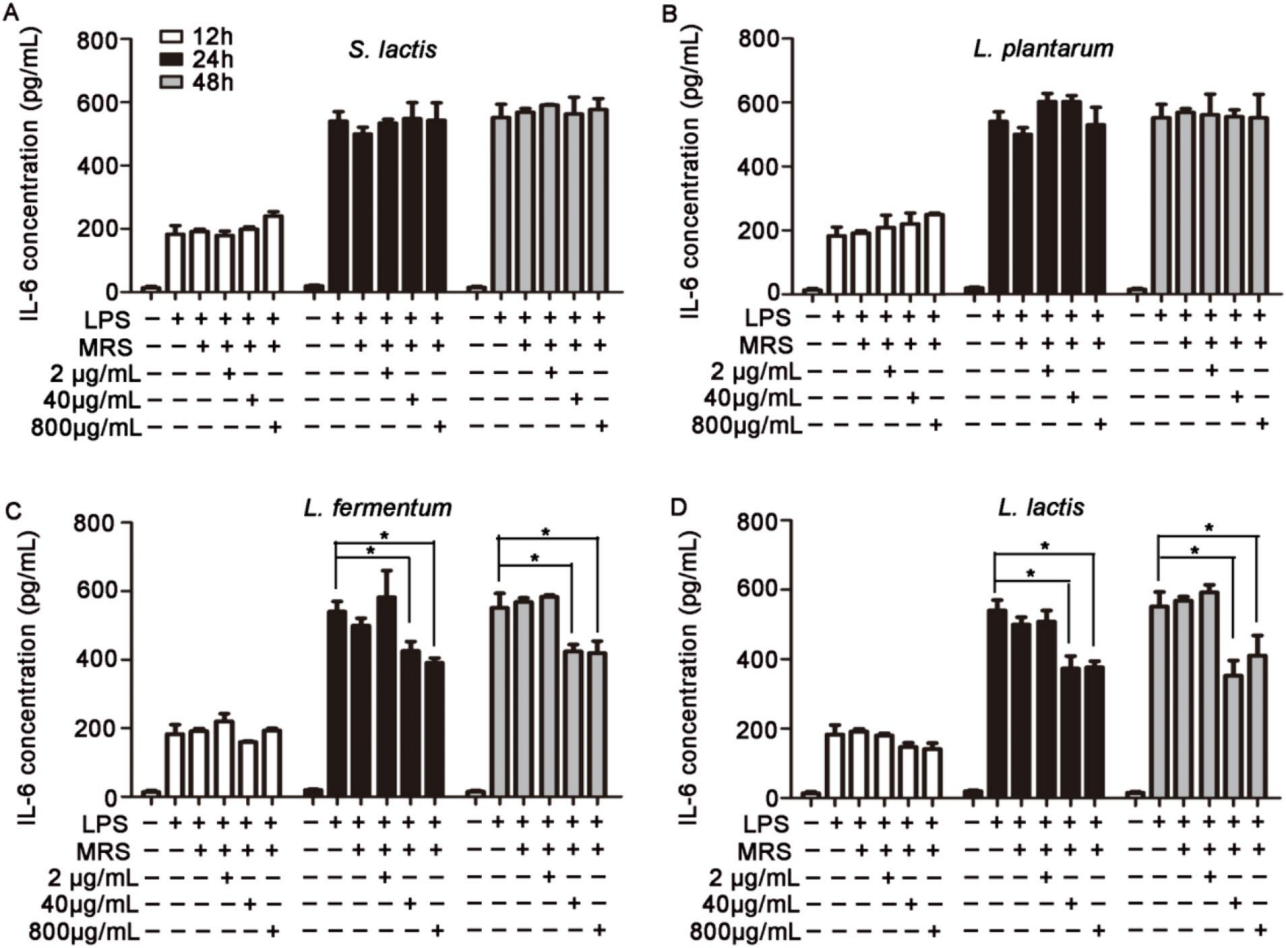
Effects of extracellular products of probiotics on TNF-α, IL-6, and IL-8 levels in HUVECs. **(A)** Extracellular products of *S. lactis* did not inhibit the LPS treatment-induced elevation of IL-6 levels. **(B)** Extracellular products of *L. plantarum* did not inhibit the LPS treatment-induced elevation of IL-6 levels. **(C)** Extracellular products of *L. fermentum* (40 μg/mL and 800 μg/mL) significantly reduced IL-6 levels induced by LPS treatment. **(D)** Extracellular products of *L. lactis* extracellular products (40 μg/mL and 800 μg/mL) significantly reduced IL-6 levels induced by LPS treatment (*p < 0.05).

**Figure 3:**
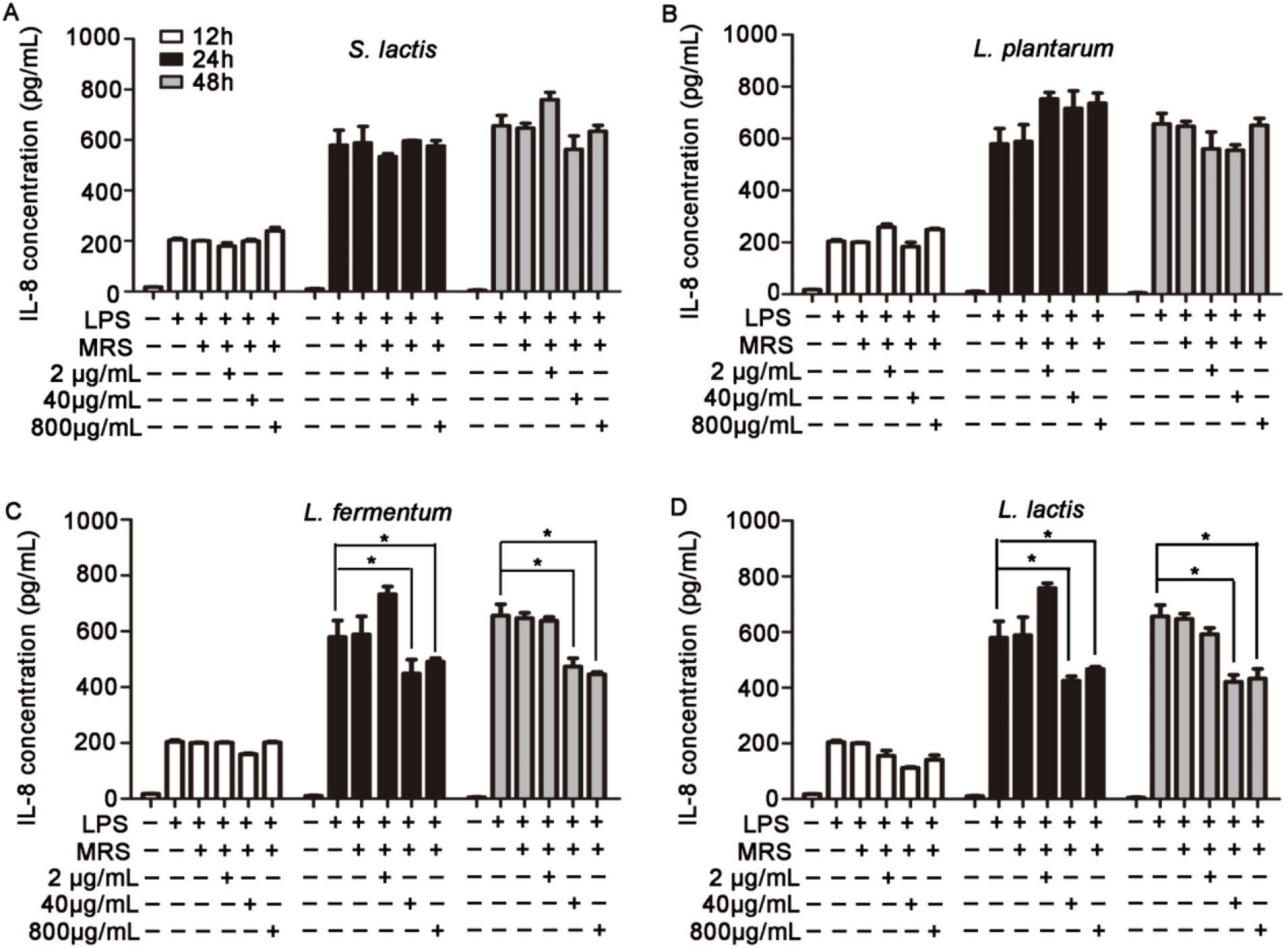
Effects of extracellular products of probiotics on TNF-α, IL-6, and IL-8 levels in HUVECs. **(A)** Extracellular products of *S. lactis* did not inhibit the LPS treatment-induced elevation of IL-8 levels. **(B)** Extracellular products of *L. plantarum* did not inhibit the LPS treatment-induced elevation of IL-8 levels. **(C)** Extracellular products of *L. fermentum* (40 μg/mL and 800 μg/mL) significantly reduced IL-8 levels induced by LPS treatment. **(D)** Extracellular products of *L. lactis* extracellular products (40 μg/mL and 800 μg/mL) significantly reduced IL-8 levels induced by LPS treatment (*p < 0.05).

HUVECs were pretreated with 2 μg/mL, 40 μg/mL or 800 μg/mL intracellular or extracellular products of *S. lactis*, *L. plantarum, L. fermentium* or *L. lactis* for 12 h, 24 h or 48 h. Then cells were cultured with 1 μg/mL LPS for 12 h. The levels of inflammatory factors in cell supernatant were detected. The results showed that the intracellular products of these four probiotics could not inhibit the increased levels of IL-6 and IL-8 induced by the LPS treatment (Supplementary Figures S3, S4). But, after the cells were treated with extracellular products of *L. fermentium* or *L. lactis* (40 μg/mL and 800 μg/mL) for 24 h or 48 h, the LPS-induced levels of IL-6, IL-8 and TNF-α were reduced significantly (Figures 2, 3; Supplementary Figure S5). These data suggested that pretreatment of cells with extracellular products of *L. fermentium* or *L. lactis* for 24 h or 48 h inhibited the levels of inflammatory cytokines induced by LPS. Thus, *L. lactis* was selected for the further experiments *in vivo*.

### 3.4 Effects of extracellular products of *Lactococcus lactis* on the mortality rate of mice

After 7 days treatment with MRS medium or extracellular products of *L. lactis*, the mice were intraperitoneally injected with 20 mg/kg LPS to establish the sepsis model. Firstly, the mortality rate of mice was observed. The mortality rate in the Mock group was 0%. The mortality rate increased to 82% in the LPS group. The MRS medium could not inhibit the mortality rate induced by LPS. But after pretreated with 0.2 g/kg or 0.4 g/kg extracellular products of *L. lactis*, the mortality of mice was decreased significantly (Supplementary Figure S6). Thus, the mortality of sepsis might be inhibited by extracellular products of *L. lactis* pretreatment.

### 3.5 The effects on the levels of TNF-α and IL-6 in the serum of mice

**Figure 4:**
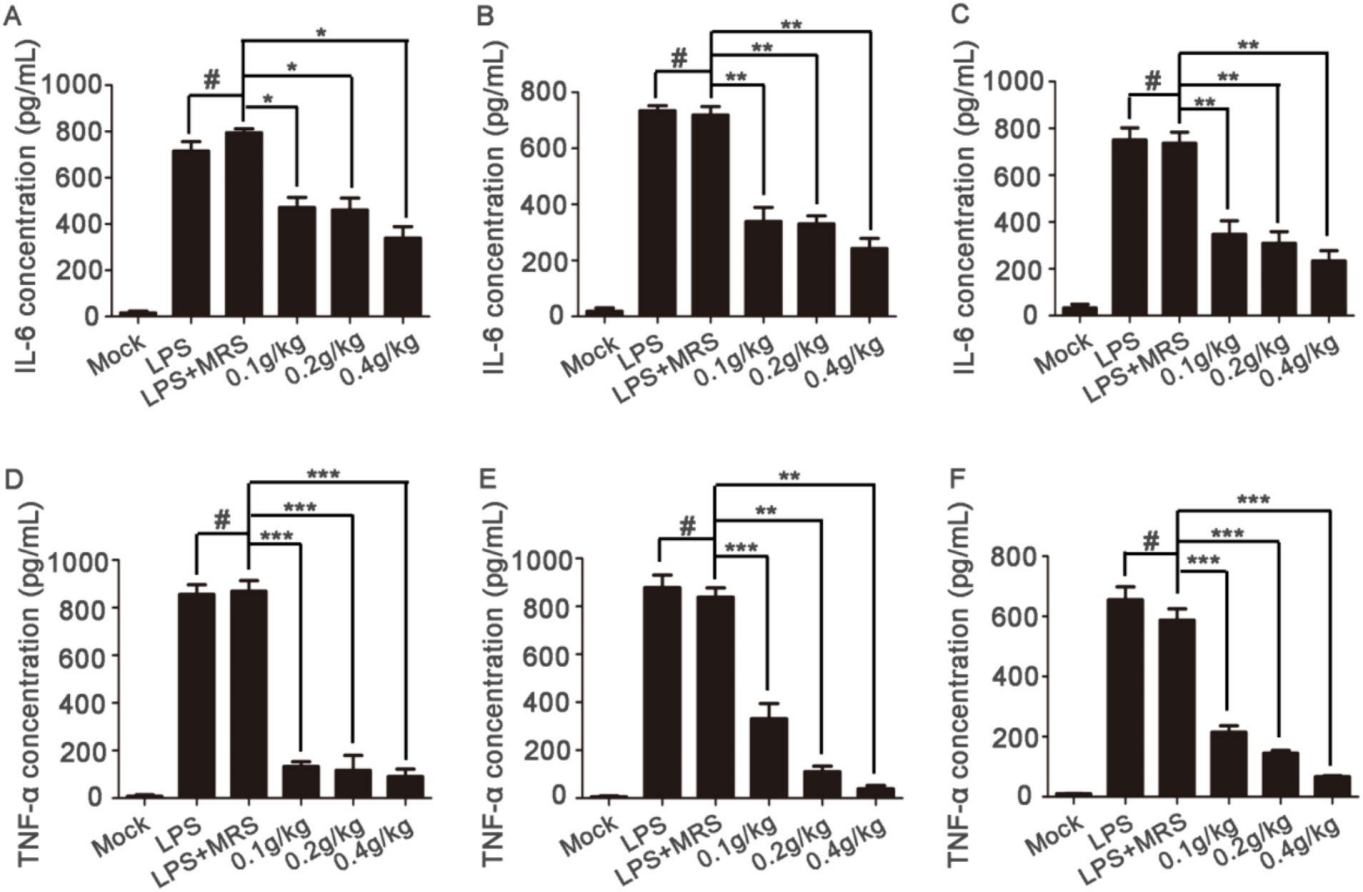
Serum levels of inflammatory factors in mice Serum levels of IL-6 were detected after blood collection at 6 h, 12 h, and 24 h after LPS injection **(A–C)**; serum levels of TNF-α were detected after blood collection at 6 h, 12 h, and 24 h after LPS injection **(D–F)** (*p < 0.05, **p < 0.01, ***p < 0.001) (“0.1 g/kg” is the abbreviation of “LPS + 0.1 g/kg extracellular products of *L. lactis*,” “0.2 g/kg” is the abbreviation of “LPS + 0.2 g/kg extracellular products of *L. lactis*,” “0.4 g/kg” is the abbreviation of “LPS + 0.4 g/kg extracellular products of *L. lactis*”).

Blood samples were collected at 6 h, 12 h or 24 h after LPS injection to detect the levels of TNF-α and IL-6 in serum. After pretreatment with extracellular products of *L. lactis,* the increased IL-6 and TNF-α levels induced by LPS in the serum of mice was significantly decreased compared with those in the LPS + MRS groups. And the high concentration showed the best effects (Figure 4). These results suggested that the extracellular products of *L. lactis* could inhibit the levels of IL-6 and TNF-α in the serum of mice induced by LPS.

### 3.6 Organ coefficient and lung index

**Figure 5:**
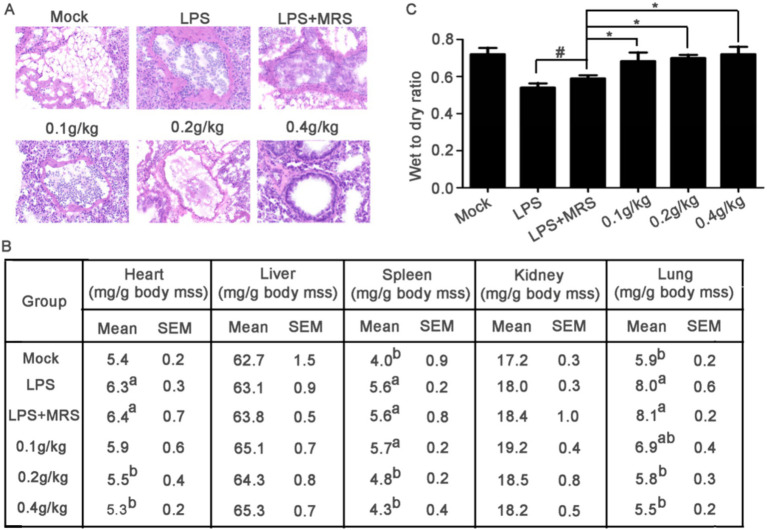
Mouse sections and organ coefficients. **(A)** Pathological changes of lung tissue in mice stained by HE. **(B)** Comparison of heart, liver, spleen, kidney, and lung coefficients among different groups of mice (^a^ compared with the mock group *p* < 0.05; ^b^ compared with LPS group, *p* < 0.05). **(C)** Assessment of pulmonary edema by evaluating the wet-to-dry ratio of mouse lungs (*p < 0.05).

Lung lesion is one of the main symptoms of acute sepsis in mice. The H&E staining data showed that in the Mock group, the lung tissue structure was complete, the alveolar structure was clearly visible and no inflammatory cell infiltration was observed in the alveoli. In the LPS group and LPS + MRS group, the alveolar cavity morphology was severely deformed, the alveolar wall was significantly thickened and there were a large number of inflammatory cell infiltration in the alveolar and the alveolar wall. In the three extracellular products of *L. lactis* treatment groups, the number of inflammatory cell infiltration in the alveoli was significantly reduced, the alveolar wall became thinner and the shape of the alveolar cavity gradually recovered. With the increased concentration, the recovery of lung injury gradually improved. In the high concentration group (0.4 g/kg), the morphology was similar to that in the Mock group (Figure 5A).

After LPS stimulation, the heart, spleen and lung coefficients were significantly increased compared with that in Mock group. All these three organ coefficients in LPS + MRS groups were significantly increased compared with those in Mock groups, but there were no significant differences with LPS groups, indicating that MRS pretreatment could not change the increased heart, spleen, and lung coefficients induced by LPS. Compared with LPS + MRS Group, the heart, spleen, and lung coefficients in *L. lactis* extracellular products treated groups were significantly decreased, suggesting that pretreated with *L. lactis* extracellular products could effectively prevent the heart, spleen, and lung damage induced by LPS (Figure 5B).

Pulmonary edema is an important phenomenon of sepsis in mice. The results showed that the wet to dry ratio of lung was significantly decreased after LPS stimulation in mice, indicating that 20 mg/kg LPS stimulation could significantly cause the symptoms of pulmonary edema in mice. There was no significant difference in the wet to dry ratio between the LPS + MRS group and the LPS group, indicating that MRS could not improve the symptoms of pulmonary edema. Compared with LPS or LPS + MRS group, not only the lung coefficient in the low/medium/high dose groups was significantly decreased, but also the lung wet to dry ratio was significantly decreased which indicated that the *L. lactis* extracellular products could effectively improve the symptoms of pulmonary edema induced by LPS (Figure 5C).

### 3.7 Enrichment analysis of the gene ontology (GO) terms of the DEGs functional

To find the mechanisms that *L. lactis* extracellular products contribute to the sepsis prevention, we analyzed extracellular products of *L. lactis*-induced changes in the HUVEC gene expression profile treated with LPS by using Genome Array. Compared to normal and LPS treated HUVECs, 981 genes had more than 2-fold changes in HUVECs, including 420 genes up-regulated and 561 genes down-regulated (Supplementary Figure S7A). Compared to LPS and sample treated HUVECs, 50 genes had more than 2-fold changes in HUVECs, including 14 genes up-regulated and 36 genes down-regulated (Supplementary Figure S7B).

**Figure 6:**
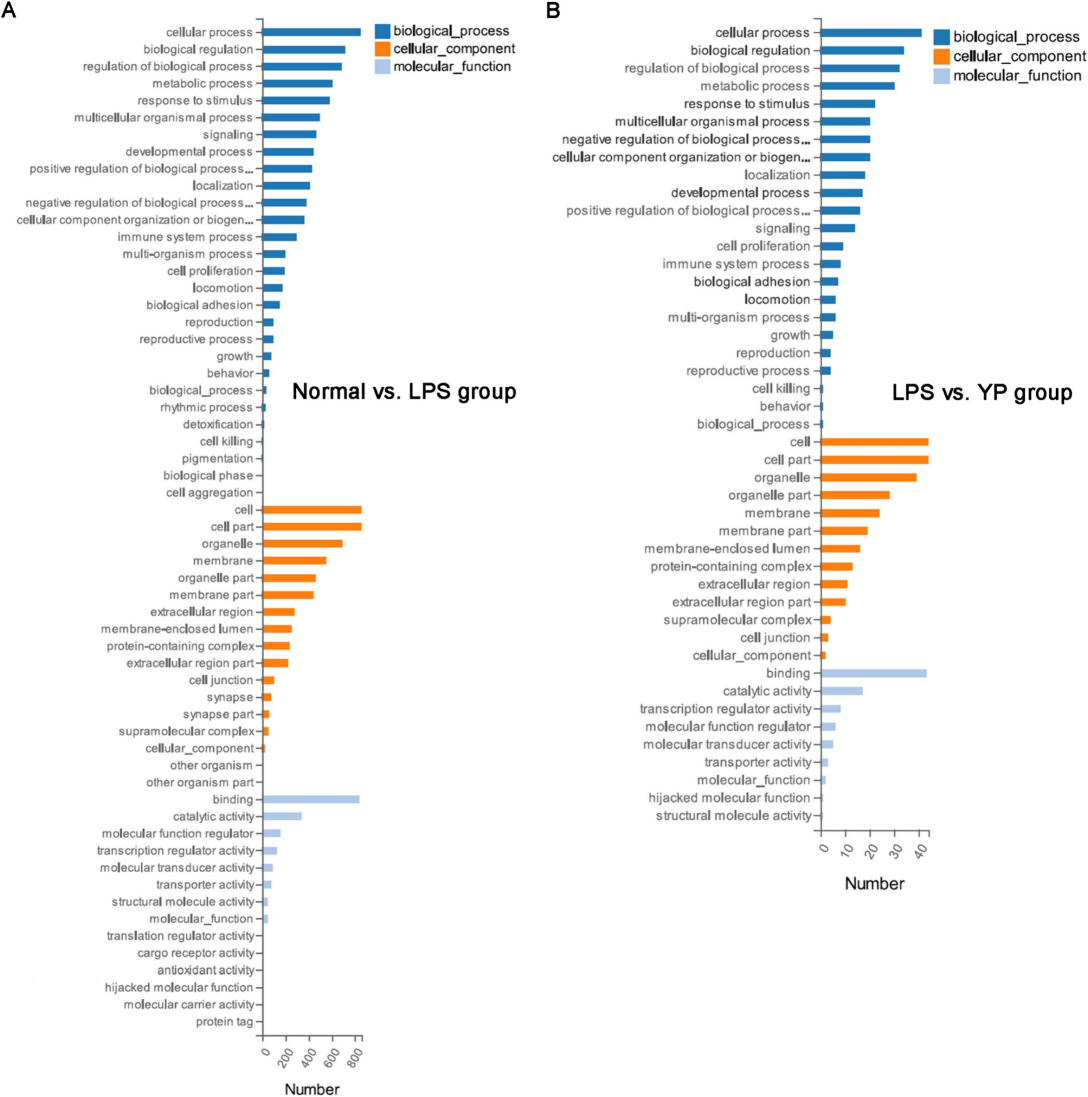
GO analysis of DEGs. **(A)** Normal group was compared with LPS group. **(B)** The LPS group was compared with the sample group (“YP group” means “LPS+40 μg/mL extracellular products of *L. lactis*”).

GO enrichment analysis was performed for DEGs. The GO enrichment histogram directly showed the quantity distribution of different expressed genes in biological processes, cell components and molecular functions (Figure 6). Compared with the normal group, most DEGs in the LPS group were related to cellular process and metabolic process in the biological processes category. In the cellular component category, most are related to the cell, cell part and organelle part. In the molecular function category, most DEGs are associated with binding, catalytic activity and transcription regulator activity. Compared with the LPS group, most DEGs in the sample group were related to cellular process and metabolic process in the biological processes category. In the cellular component category, most are related to the cell, cell part and organelle part. In the molecular function category, most DEGs are associated with binding, catalytic activity and transcription regulator activity. In both the LPS group and the sample group, the functional changes of most DEGs, whether in biological processes, cell components, or molecular functions, were basically the same as those in the normal-LPS group, but the degree of enrichment was different.

### 3.8 Kyoto encyclopedia of genes and genomes pathway analysis of DEGs

**Figure 7:**
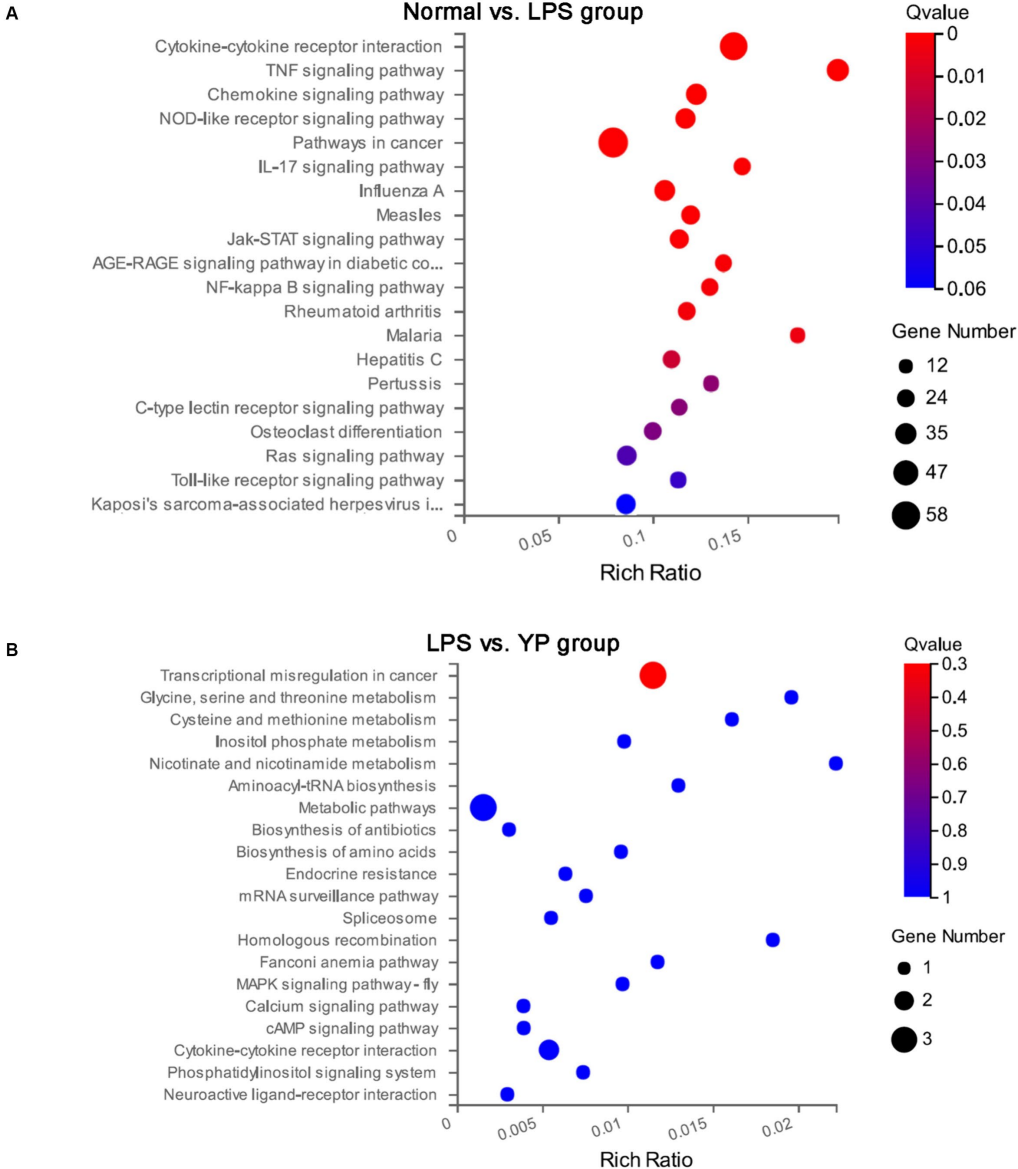
Kyoto Encyclopedia of Genes and Genomes (KEGG) pathway analysis of DEGs. **(A)** Normal group was compared with LPS group. **(B)** The LPS group was compared with the sample group.

The top 20 significant pathways of DEGs were selected and represented in KEGG enrichment scatter diagram (Figure 7). In the normal-LPS group, DEGs were mainly enriched in cytokine-cytokine receptor interaction and pathways in cancer. DEGs were also enriched in cytokine—cytokine receptor interactions compared with the sample-LPS group. In the normal-LPS group, DEGs were enriched in inflammatory factor-related pathways, such as TNF signaling pathway and IL-17 signaling pathway, but in the sample-LPS group, no inflammatory factor-related pathway genes were enriched.

## 4 Discussion

Sepsis is a clinical syndrome characterized by systematic inflammation and circulatory malfunctions following the pathogenic infection like bacteremia or fungemia. It is one of the most common causes of morbidity and mortality in critically ill patients (Yoon, 2012) and preterm infants. In addition, sepsis is associated with significant morbidity and reduced quality of life among sepsis survivors (Oud, 2014). Probiotics have been explored in an exponentially increasing number of clinical trials for their health effects (Jansen et al., 2021; DeVeaux et al., 2023). The current reports suggested that the probiotics use is associated with a 36.9% reduction in the incidence of postoperative sepsis (Arumugam et al., 2016). Since supplementation of probiotics significantly reduces the incidence of postoperative sepsis without significant negative side effects, these probiotic supplements may be considered as an effective and potentially routine treatment for patients undergoing gastrointestinal surgery (Kim et al., 2019; Potruch et al., 2022). Probiotic translocation might result in some clinical infections, including systemic and localized infections (María Remes-Troche et al., 2020). Predominantly, infections associated with specific probiotics include sepsis. The ingestion of live microorganisms (probiotics) in rare instances might surplus the risk of sepsis as evidenced by several studies, raising the safety concern of routine probiotic use in such population. Nevertheless, growing pieces of evidence support the beneficial role of probiotics in critically ill and neonates (Litao et al., 2018). Maintenance or restoration of intestinal microbiota and metabolite composition may be a therapeutic or prophylactic target for sepsis (Potruch et al., 2022). Our results suggested that *Lactococcus Lactis* metabolites could reduce inflammatory factors in cells and blood of mice which might be useful to prevent sepsis.

The endothelial cell lining (ECL) of the vasculature is a unique cellular system that coats the inside of blood vessels and forms the interface between the circulating blood and the parenchymal cells responsible for organ function (Kim et al., 2013). It is critical for the regulation of hemostasis, vasomotor control, and immunological function, by sensing and reaction through secretion of molecules, which initiate transcellular and intracellular signaling (Cheng and Huang, 2021). In addition to these important functions, the endothelium forms the essential vascular barrier for solute transport and osmotic balance. Sepsis is associated with severe endothelial cell (EC) dysfunction leading to dysregulation of hemostasis and vascular reactivity, as well as tissue edema (Dalan et al., 2017). This failure of the ECL is considered central to the progression to organ failure during sepsis (Beltrán-García et al., 2022). The glycocalyx is a gel-like layer lining the luminal surface of endothelial cells, the degradation of glycocalyx is also thought to contribute to microcirculatory dysfunction in sepsis (Wu et al., 2017). Many preclinical and clinical studies have demonstrated an association between inflammatory cytokines such as TNF-α, IL-1β, IL-6, and IL-10 and glycocalyx degradation biomarkers (Gao et al., 2018; Tamura et al., 2020). Our results showed that LPS-induced levels of IL-6, IL-8, and TNF-α were significantly decreased after treatment with extracellular products of *L. fermentium* or *L. lactis* (40 μg/mL and 800 μg/mL) for 24 or 48 h. These data indicated that the extracellular products of *L. fermentium* or *L. lactis* inhibited LPS-induced inflammatory cytokine levels.

Our results showed that the extracellular products of *L. fermentium* or *L. lactis* inhibited LPS-induced inflammatory cytokine levels. The function of extracellular products of *L. fermentium* has been published before (Wang et al., 2023). We tested the preventative effects of extracellular products of *L. lactis* using an LPS-induced mouse sepsis model. The lungs have been shown to be the most susceptible organ to sepsis, and protecting the lungs from inflammation is considered a promising strategy for treating sepsis (Wang Z. F. et al., 2020; Ling et al., 2023). Histopathological analysis showed that extracellular products of *L. lactis* relieved inflammatory cell infiltration and pulmonary edema in lungs treated with LPS. Additionally, it improved survival rate and inhibited serum proinflammatory cytokine levels in mice. This suggested that extracellular products of *L. lactis* could improve LPS-induced septicemia by reducing the severity of systemic inflammation in lung.

Because the pathogenesis and treatment of sepsis are still unclear, clinical treatment brings great burden to society and patients. The search for potential biomarkers is important for future sepsis treatment and prognosis. Studies have shown that the host response of sepsis does not conform to the simple high/low inflammation model (Jones et al., 2022).

Mitochondria are not only key regulators of metabolite biosynthesis, but also play important roles in many other cellular functions, such as calcium homeostasis, hormone metabolism, thermoregulation, production of reactive oxygen species and nitrogen, and cellular signaling (Ciron et al., 2012; Sharma et al., 2018). GO analysis showed that most of the DGEs were enriched in the cellular structure and metabolic processes in both the normal-LPS group and the LPS-sample group. It has been reported that sepsis easily mitochondrial damage and impaired energy metabolism in humans, which may be an important part of sepsis leading to multiple organ dysfunction syndrome (Liu et al., 2023; Yang et al., 2023). Therefore, resuscitating metabolism and restoring cellular structure through the mitochondrial pathway would be a new cornerstone of sepsis therapy.

GO analysis of DEGs showed that the functional changes of DEGs in the normal-LPS group all included the functional changes of DEGs in the lPS-sample group, indicating that the 50 significant genes in the lPS-sample group were most likely contained in the 981 significant genes in the LPS-sample group. It is highly likely that the sample can prevent sepsis by regulating these 50 significant genes. Interestingly, other articles have found that regulating cytokines and genes associated with immune responses to LPS can reduce the symptoms of LPS-induced sepsis in mice, which is consistent with our study. in addition, in KEGG analysis, samples can affect Transcriptional misregulation in cancer related genes (Rosier et al., 2022; Chen et al., 2024) Our study showed that samples can reduce the secretion of pro-inflammatory cytokines in sepsis mice. Whether it is related to Transcriptional misregulation in cancer related genes still needs to be further explored (Rawat et al., 2021).

Granulocyte-macrophage colony-stimulating factor (GM-CSF), a hematopoietic growth factor, is currently used in patients with neutropenia induced by myelosuppression in chemotherapy to counteract the immunosuppression of sepsis (Mathias et al., 2015; Venet and Monneret, 2017). Lipopolysaccharides play a crucial role in activating the immune system, in particular by acting on systemic immune-activated cells to stimulate the production of pro-inflammatory cytokines, and the investigated Immunostimulatory therapies alone include GM-CSF, and although it has been shown to improve clinical indicators, pro-inflammatory therapies aimed at preventing sepsis are currently being investigated (Maldonado et al., 2016; Hall, 2019). Meanwhile, lipopolysaccharides are able to activate neutrophils *in vitro*, as evidenced by elevated levels of TNF-α, and enhance proliferation (Mohsin et al., 2021). Our results showed that DEGs were mainly enriched in cytokine-cytokine receptor interactions in both normal-LPS and sample-LPS groups. It can be hypothesized that our samples might have an effect on sepsis through cytokine-cytokine receptor interactions, which implies that new ideas can be provided in deeper explorations in immunosuppression. However, to date, no comprehensive systematic analysis has been performed to study the complex interactions between cytokine-cytokine receptors, and this type of analysis may be needed to identify more effective therapeutic targets.

In addition, the data showed that DEGs were also associated with transcriptional dysregulation responses and inflammatory factor signaling pathways in cancer in both the normal-LPS and sample-LPS groups, which could be linked to what we talked about before, and the subtle combination of these data might play a role in the development of stress-induced immune responses, which would need to be further verified.

In summary, we finally determined that the extracellular product of *L. lactis* reduced inflammatory factors secreted by LPS-pretreated HUVECs, alleviated inflammatory cell infiltration and pulmonary edema in the lungs of septic mice, and improved the survival rate of mice, suppressed the expression level of pro-inflammatory factors within serum, and the genetic analysis preliminarily explored the pathologic mechanism, demonstrating that the extracellular product of *Lactobacillus lactis* could prevent lipopolysaccharide induced sepsis and can potentially provide new insights into the prevention of sepsis at the molecular level.

## 5 Conclusion

We reported that the extracellular product of *L. lactis* can effectively prevent sepsis in animal and cellular experiments, which provides more ideas for sepsis prevention methods, and more in-depth studies are needed to further explore the mechanism of protective effects of the extracellular product of *L. lactis* against LPS-induced sepsis.

## Data availability statement

The datasets presented in this study can be found in online repositories. The names of the repository/repositories and accession number(s) can be found at: https://www.ncbi.nlm.nih.gov/, 12.

## Ethics statement

The animal study was approved by all animal experimental procedures were in accordance with the laws and regulations of the People’s Republic of China and the ethical guidelines. At the same time, the procedure was carried out in accordance with the ethical guidelines and the guide for the care and use of laboratory animals issued by the National Institutes of Health. This study was approved by the Ethics Committee for Animal Experiments of Qilu University of Technology (Jinan, China). The study was conducted in accordance with the local legislation and institutional requirements.

## Author contributions

YF: Writing – original draft, Writing – review & editing, Data curation, Formal analysis. SZ: Data curation, Funding acquisition, Writing – original draft. QY: Methodology, Project administration, Writing – original draft. ZA: Resources, Supervision, Writing – original draft. MZ: Validation, Visualization, Writing – original draft. CZ: Investigation, Software, Writing – original draft. XS: Investigation, Writing – review & editing. KL: Writing – review & editing, Software. BL: Visualization, Writing–review & editing. LZ: Supervision, Writing – review & editing. LS: Software, Writing–review & editing.

## Footnotes

^1^ http://www.capitalbio.com
