# Supplementary material for: The preventative effects of Lactococcus Lactis metabolites against LPS-induced sepsis
Source: Front Microbiol. 2024 Jul 17;15:1404652. doi: 10.3389/fmicb.2024.1404652 (PMC11288810; doi:10.3389/fmicb.2024.1404652)
Supplement: Supplementary file 1 [file Data_Sheet_1.docx]

Supplementary Material

## Supplementary Figures


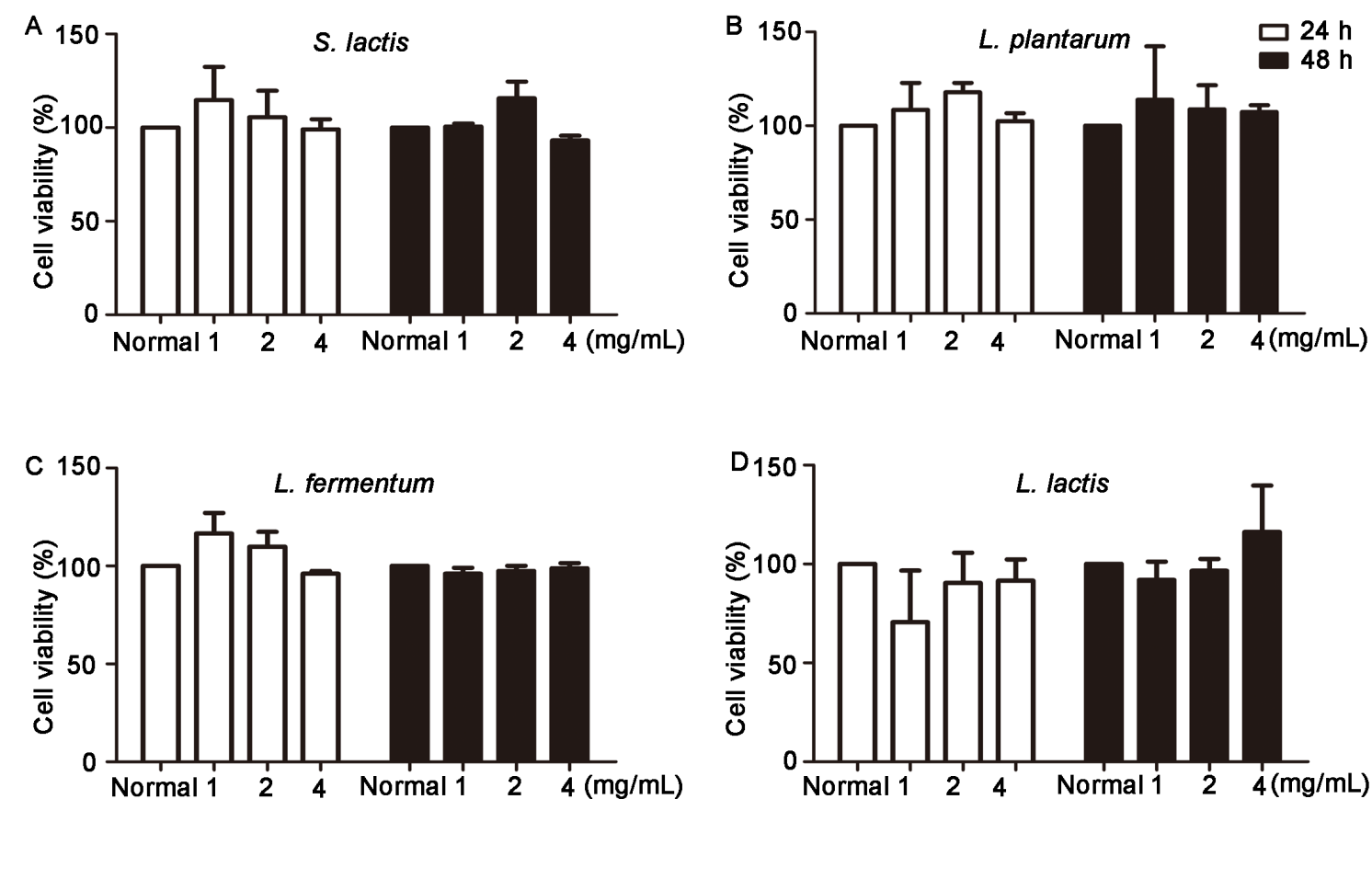


**Supplementary Figure 1 Cell viability assessment.** (A) Intracellular products of *S. lactis* do not affect cell viability at 24h and 48h. (B) Intracellular products of *L. plantarum* do not affect cell viability at 24h and 48h. (C) Intracellular products of *L. fermentum* do not affect cell viability at 24h and 48h. (D) The intracellular product of *L. lactis* does not affect cell viability at 48h.
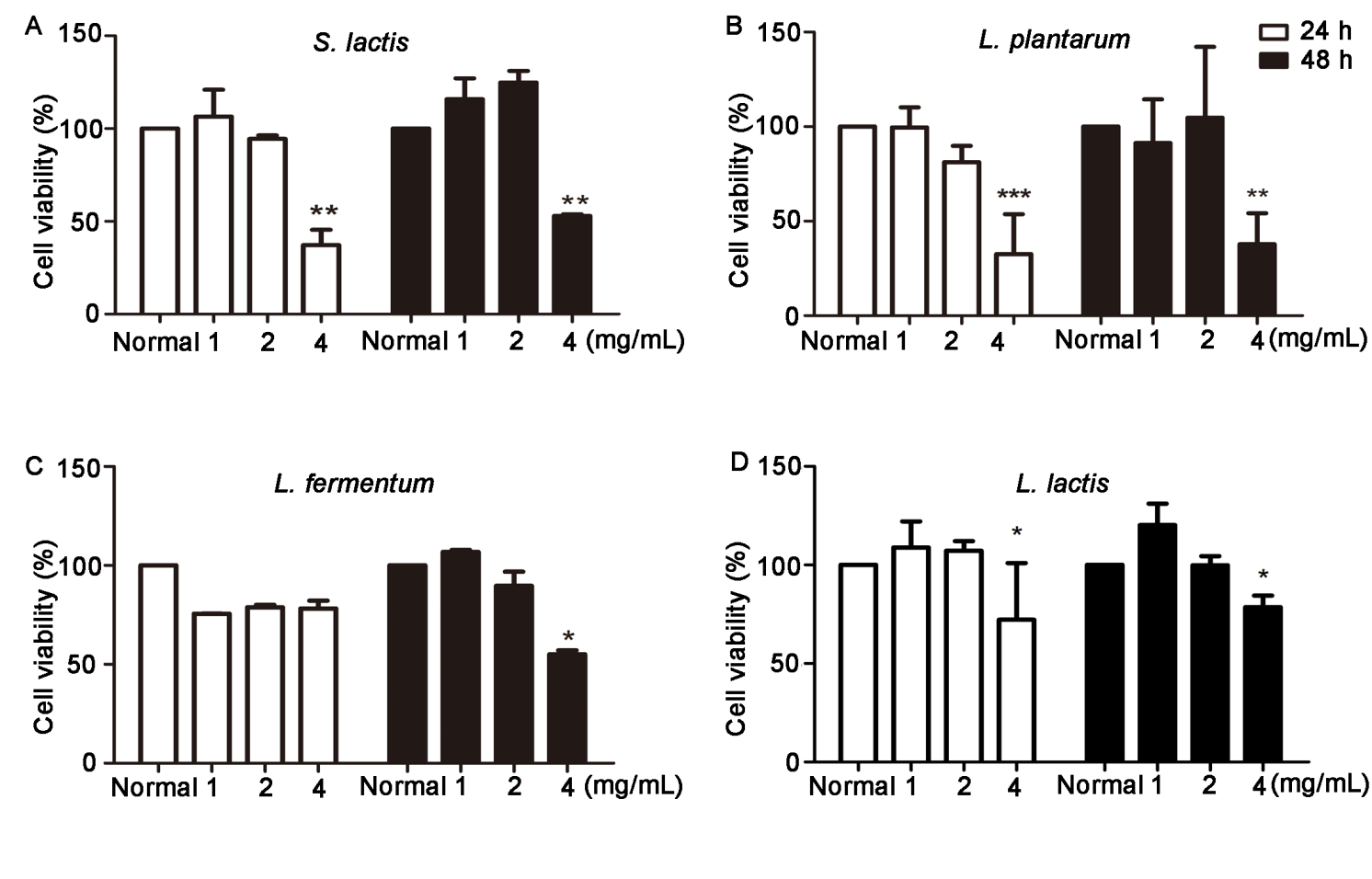


**Supplementary Figure 2 Assessment of cell viability.** (A) Extracellular products of *S. lactis* cultured cells for 24 and 48 h, and a concentration of 4 mg/ml reduced cell viability. (B) Extracellular products of *L. plantarum* cultured cells for 24 and 48h at a concentration of 4mg/ml reduced cell viability. (C) Extracellular products of *L. fermentum* cultured cells for 48h reduced cell viability. (D) Extracellular product of *L. lactis* cultured cells 24, 48h at a concentration of 4mg/ml reduced cell viability. (**p*<0.05, ***p* <0.01, ****p*<0.001)


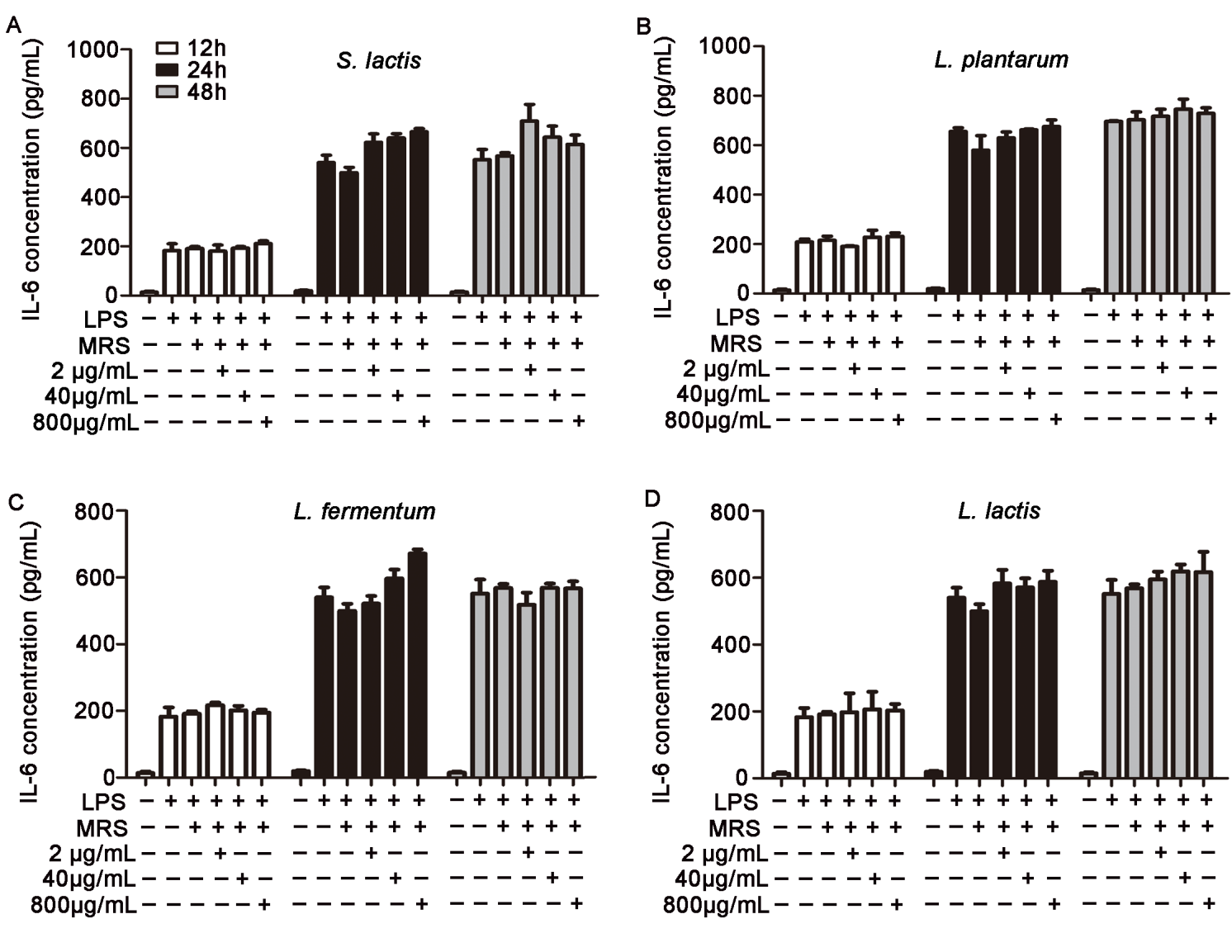


**Supplementary Figure 3 Effects of probiotic intracellular products on TNF-α, IL-6, and IL-8 levels in HUVECs** (A) Intracellular products of *S. lactis* did not inhibit LPS treatment-induced elevation of IL-6 levels. (B) Intracellular products of *L. plantarum* did not inhibit LPS treatment-induced IL-6 levels. (C) Intracellular products of *L. fermentum* did not inhibit LPS treatment-induced IL-6 levels. (D) Intracellular products of *L. lactis* did not inhibit LPS treatment-induced IL-6 levels.


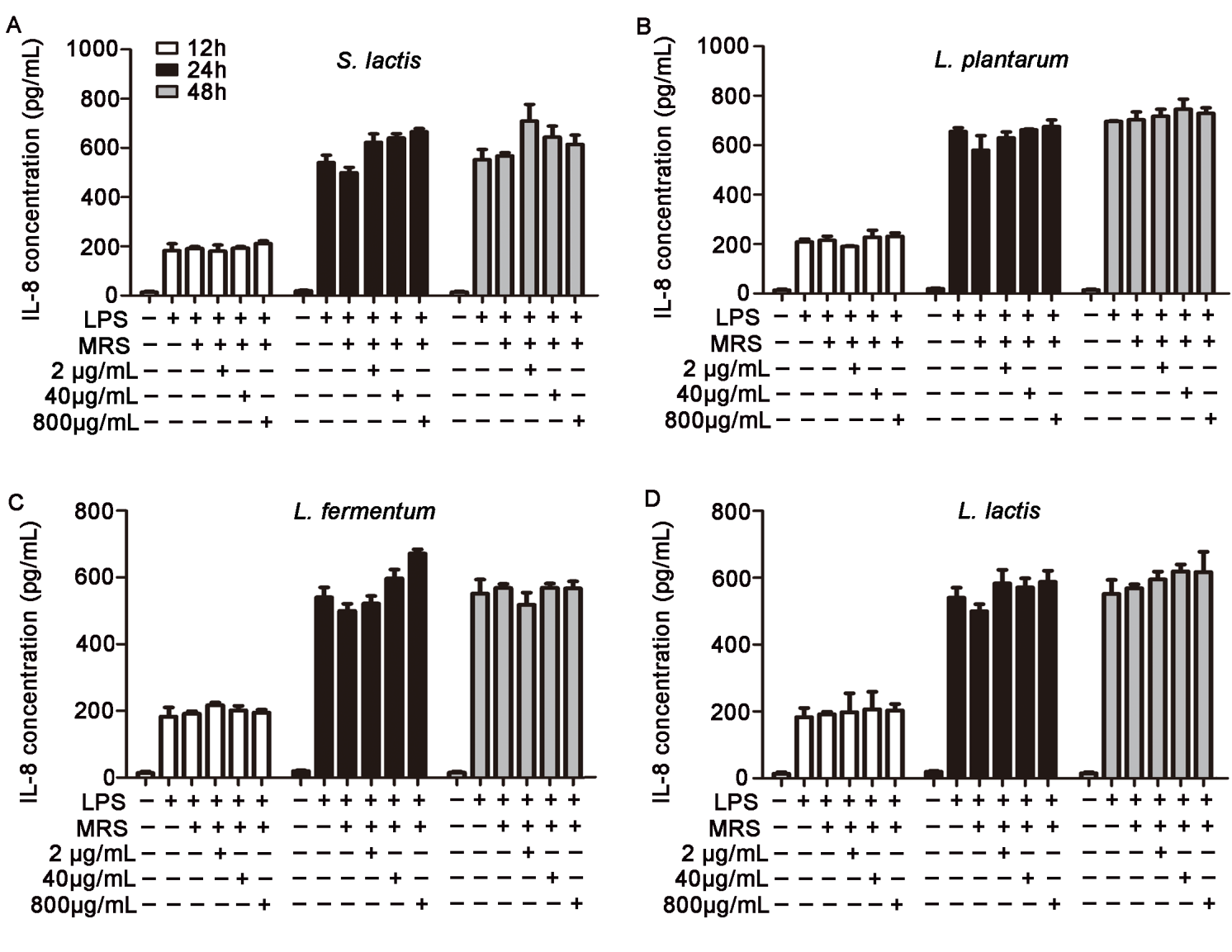


**Supplementary Figure 4 Effects of probiotic intracellular products on TNF-α, IL-6, and IL-8 levels in HUVECs.** (A) Intracellular products of *S. lactis* did not inhibit the LPS treatment-induced elevation of IL-8 levels. (B) Intracellular products of *L. plantarum* did not inhibit the LPS treatment-induced elevation of IL-8 levels. (C) Intracellular products of *L. fermentum* did not inhibit the LPS treatment-induced elevation of IL-8 levels. (D) Intracellular products of *L. lactis* did not inhibit LPS treatment-induced elevation of IL-8 levels.


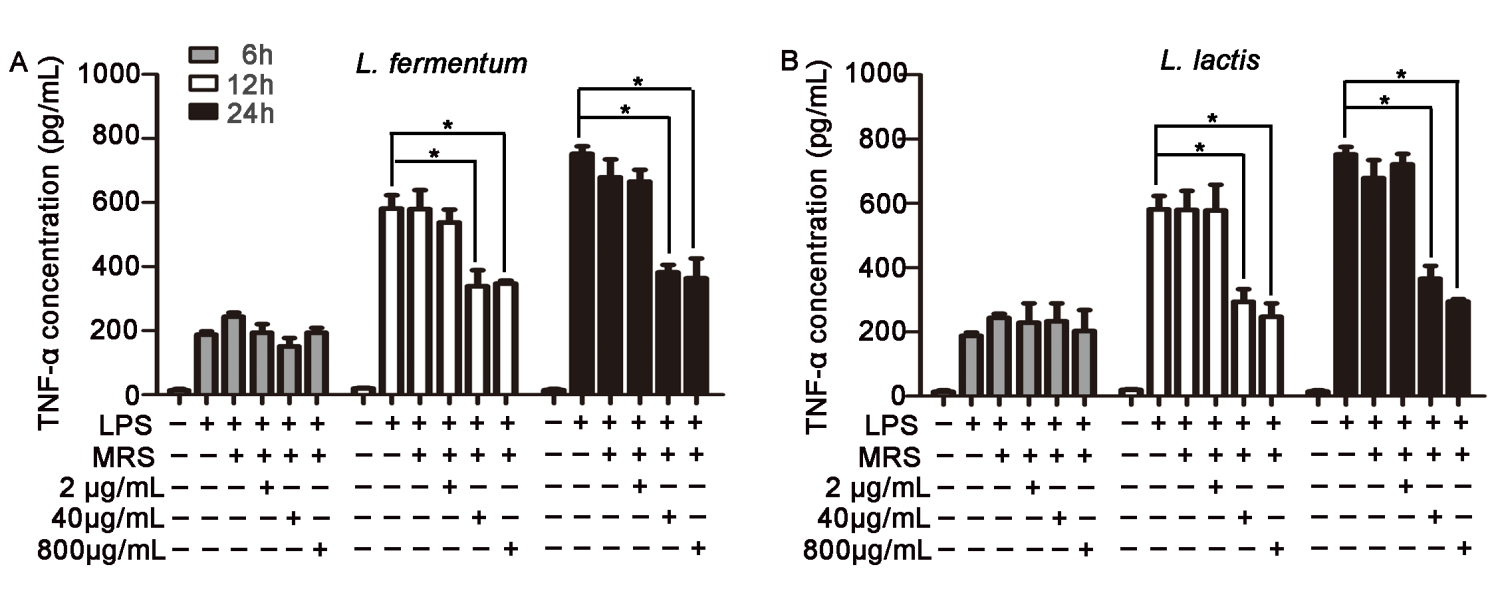


**Supplementary Figure 5 Effect of extracellular products of *L. fermentum* and *L. lactis* on TNF-α levels in HUVEC.** (A) The extracellular product of *L. fermentum* (40 μg/mL and 800 μg/mL) significantly reduced the level of TNF-α induced by LPS treatment. (B) The extracellular product of *L. lactis* (40 μg/mL and 800 μg/mL) significantly reduced the level of TNF-α induced by LPS treatment. (**p*<0.05)


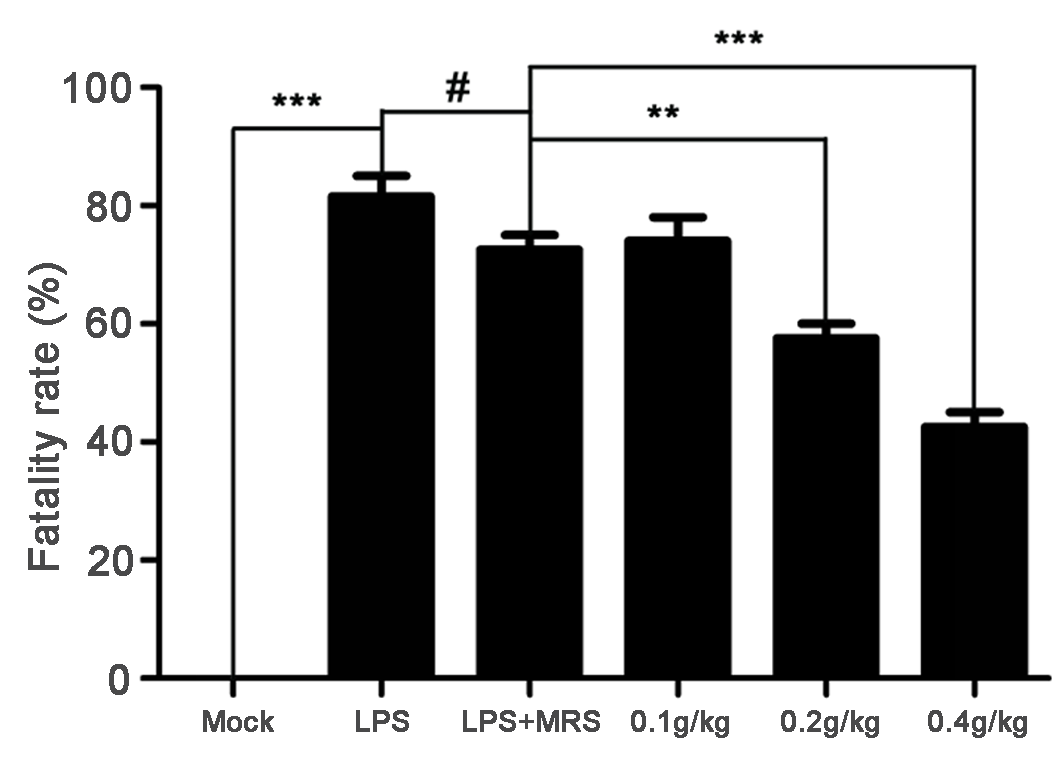


**Supplementary Figure 6 Effect of extracellular products of *L. lactis* on mortality of mice.** (***P* <0.01, ****P*<0.001)


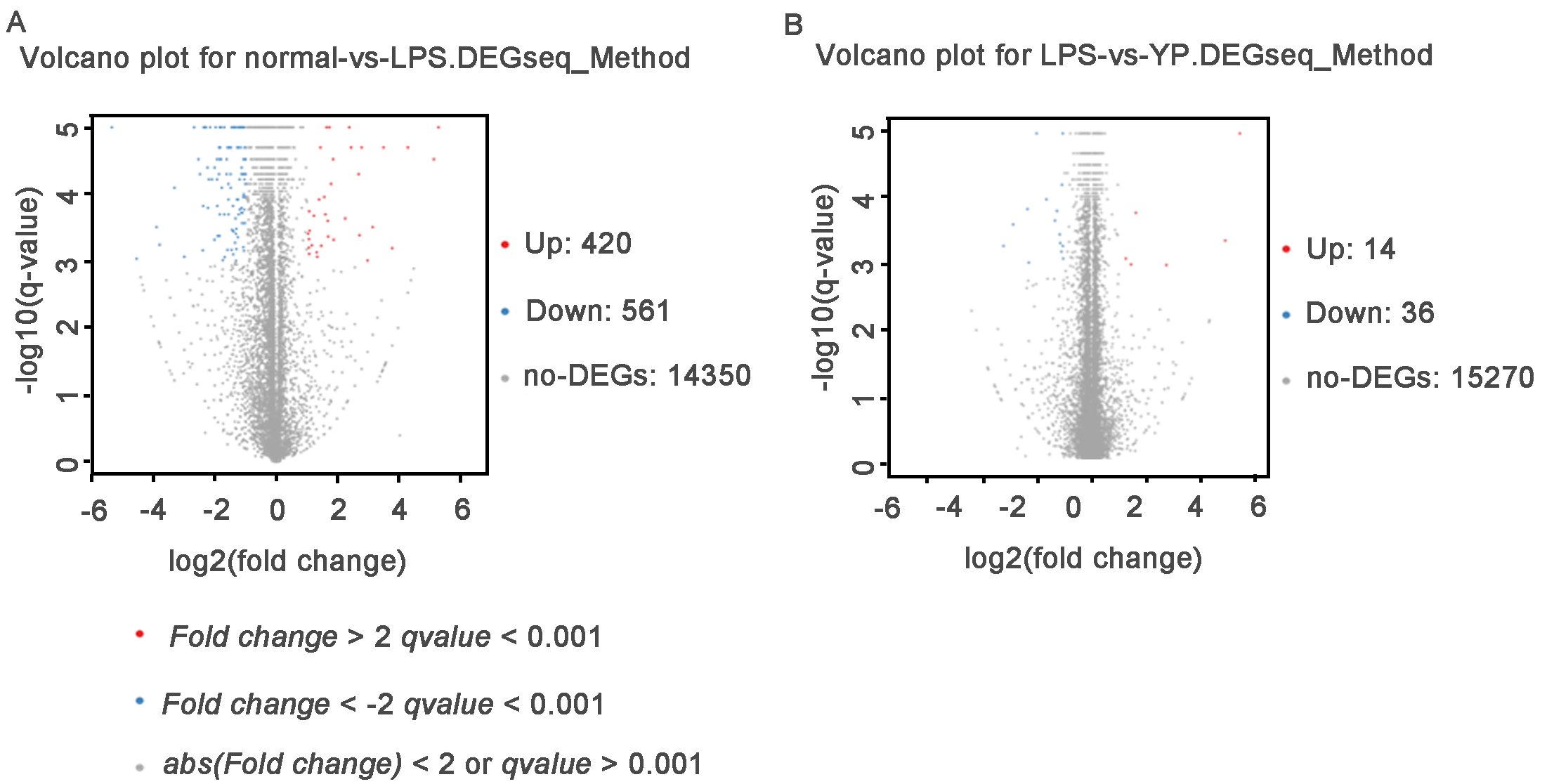


**Supplementary** **Figure 7 Volcano diagram of gene chip.**
